# Increasing Point-of-care Ultrasound Scanning by Pediatric Emergency Medicine Fellows: A Quality Improvement Initiative

**DOI:** 10.1097/pq9.0000000000000854

**Published:** 2025-11-28

**Authors:** Rosemary Thomas-Mohtat, Ashley Booth, Alia Fink, Eva I. Rubio

**Affiliations:** From the * Division of Pediatric Emergency Medicine, Children’s National Hospital, Washington, D.C.; † George Washington School of Medicine and Health Sciences, Washington, D.C.; ‡ Department of Quality & Safety, Children’s National Hospital, Washington, D.C.; § Division of Pediatric Radiology, Starship Children’s Hospital, Auckland, New Zealand.

## Abstract

**Introduction:**

During the past 20 years, there has been an increased integration of point-of-care ultrasound (POCUS) curricula in pediatric emergency medicine (PEM) fellowships. Locally, our fellows were not achieving the recommended 150 scans target we set for graduation. We undertook this quality improvement initiative to increase POCUS scanning in our PEM fellowship.

**Methods:**

We implemented a quality improvement project from July 2020 to June 2021. Our primary aim was to increase the number of scans performed monthly by the fellows from 3 to 5 scans per fellow per month. After querying the fellows and identifying barriers to POCUS scanning, we implemented interventions targeted at increasing longitudinal scanning, transparency, and accountability in the achievement of target scan numbers.

**Results:**

The number of scans per fellow per month increased from a baseline of 3 to 7.6 scans per fellow per month. All graduating fellows met the target of 150 scans by graduation. The impact was sustained 5 years later.

**Conclusions:**

The most effective interventions included required quarterly supervised scanning, maintaining a transparent scan-count dashboard, and encouraging scanning with co-fellows when working clinically. These strategies can be incorporated by other PEM training programs.

## INTRODUCTION

Point-of-care ultrasound (POCUS) has undergone a 20-year evolution in pediatric emergency medicine (PEM) with curricular development that has resulted in its inclusion in the Accreditation Council for Graduate Medical Education core PEM training requirements, testable content on the American Board of Pediatrics Board examination for PEM, a PEM POCUS–specific research network, and PEM POCUS fellowships.^1–8^ Despite these advances, multiple studies during the past 5 years have cited inconsistencies in POCUS curricula, potentially resulting in suboptimal use of POCUS as a skill upon graduation from PEM fellowship training.^9,10^ Nearly all training programs have some kind of curriculum; however, there is variability in the curriculum content and implementation, acquisition and monitoring of skills, and time commitment to hands-on scanning.^9,10^ Acuña et al^11^ cited the most common barriers to POCUS competency as the lack of qualified faculty available for training (62.9%), lack of confidence or comfort in using the existing ultrasound machine(s) in their department (54.8%), and physician resistance to using new technology (50%). Gold et al^10^ reported different barriers: discomfort with POCUS skills (76.7%), insufficient educational time to learn POCUS (65%), and perceived negative impact of POCUS on efficiency (58.5%).

POCUS is a procedural skill and requires continued practice to maintain fluency. Fluency in ultrasound requires 2 crucial elements: comfort with its mechanical performance and confidence in the interpretation of the imaging obtained.^12^ According to motor learning theory, the frequency of training has a far greater effect on the retention of motor memory than the duration of a single training.^13^ Regarding interpretation, Boutis et al^14^ noted that frequent exposure is necessary to decrease the learning decay of discrete interpretation skills. Interpreting POCUS images is complex, especially with the multiple different applications that are practiced. A 2023 cross-sectional survey of recent PEM fellowship graduates reported on the use of POCUS by pediatrics-trained PEM fellows. They were using POCUS less frequently and in fewer applications upon graduation than the emergency medicine (EM)–trained PEM fellows or PEM fellows who went on to complete additional POCUS fellowship.^15^

In their 2001 guidelines, the American College of Emergency Physicians (ACEP) recommended the performance of a minimum of 150 scans during 3 years for EM physicians seeking general emergency ultrasound privileges.^16^ Therefore, in our PEM fellowship program, we targeted 150 scans across all POCUS applications. However, our PEM fellows were falling short of the recommended 150 scans at graduation.

We undertook this quality improvement (QI) project to address barriers to POCUS use in a PEM fellowship setting. Our SMART aim was to increase longitudinal POCUS scanning by PEM fellows from 3 to 5 scans per fellow per month by June 2021 and to sustain for 12 months. This targeted improvement would represent an increase from a baseline of 90 scans per fellow completed during 3 years to the goal of 150 scans per fellow after the initiative. Ultimately, the global aim of our QI initiative was to prepare the next generation of PEM physicians to use POCUS in clinical decision-making to impact patient care.

## METHODS

The baseline period for this QI initiative was from July 2018 to June 2019. The baseline period was chosen as the year preceding the interventions because the COVID-19 pandemic started during the academic year from July 2019 to June 2020, and all ultrasound activities were halted for the first 4 months of the COVID-19 pandemic (March–June 2020). The interventions occurred from July 2020 through June 2021.

## CONTEXT

### Setting

Our POCUS program operates at 2 clinical sites—a quaternary care, free-standing children’s hospital and a community site with a separate pediatric ED and an ultrasound machine dedicated to POCUS at each site. In the baseline period, annual patient visits were 125,326 across the 2 sites. In the intervention period (which was during the beginning of the COVID-19 pandemic), the pediatric volumes in the ED were significantly decreased to 72,707 annual ED visits. At the time of the QI intervention, our ultrasound program had 4 POCUS fellowship–trained PEM faculty and 7 additional PEM faculty credentialed in POCUS through a practice pathway. The practice pathway allows PEM faculty to be credentialed in POCUS if they completed an adequate number of studies as outlined by ACEP ultrasound credentialing guidelines in specific applications (such as 25 cardiac examinations with 5 positive studies).^17^ This group contrasts with POCUS fellowship–trained faculty who complete 1,000 ultrasounds and have additional training in quality assurance (QA) and image review. In the baseline period, the PEM fellowship program consisted of 4 fellows each year. In 2020, the fellowship size increased to 5 fellows a year.

### Baseline Ultrasound Curriculum

During fellowship orientation, first-year fellows attend a 2-day POCUS boot camp with the EM intern class from our affiliated university. In the first 3 months of their fellowship, they have 5 scan sessions with 1 QA session. Scan sessions are dedicated 3-hour sessions without assigned patient responsibilities during which fellows scan patients assigned to other physicians, with the scans deemed either educational or assisting with medical decision—making (MDM). During their second year, fellows have a dedicated POCUS rotation with at least 10 ultrasound scan sessions, with up to 4 learners at a time. All scan sessions are supervised by POCUS fellowship–trained faculty and are 3 hours in duration. During their final year of training, there is no dedicated ultrasound time, but the fellows are encouraged to scan on their own. In addition, the fellows have accounts for ImageSim (Toronto, Canada), a competency-based e-module program for image interpretation in 4 specific applications (Soft Tissue, Focused Assessment with Sonography in Trauma [FAST], Cardiac, and Lung); they are required to achieve competency in all 4 modules. POCUS applications include Cardiac, FAST, Biliary, Soft Tissue, Lung, Bowel, Renal, Bladder, Musculoskeletal, Obstetrics/Pelvic, Procedural, Ocular, Scrotal, Neonatal Head, and Vascular.

### QA Process

All images are automatically transmitted to our image archiving database (OsiriX, Geneva, Switzerland). This database facilitates the review of all saved examinations for QA without requiring manual submission by the fellows. POCUS fellowship–trained faculty review 100% of the scans performed from both sites. All POCUS (either educational or those used in MDM) are documented in the electronic medical record with either a procedure note when used for MDM or a specific, determined script when used only for educational purposes.

## INTERVENTIONS

**Fig. 1.**
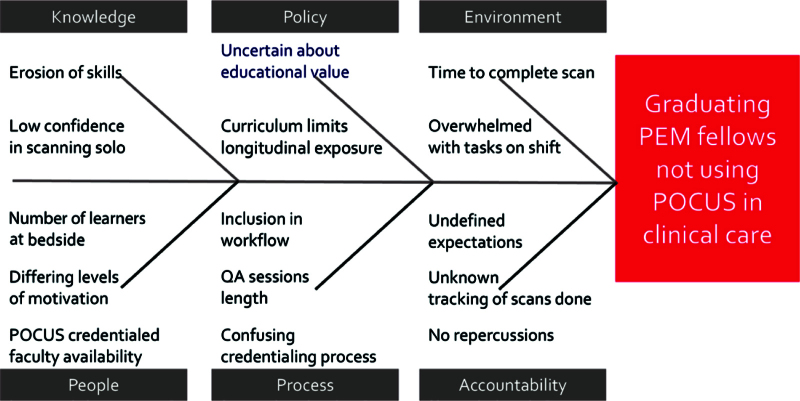
Fishbone diagram categorizing the barriers to POCUS scanning identified by PEM fellows.

**Fig. 2.**
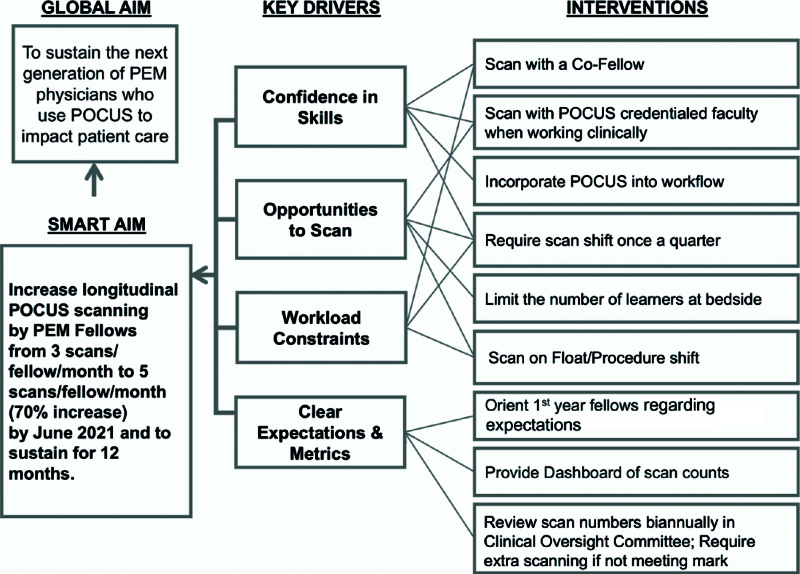
Key driver diagram for the QI initiative.

In preparation for the interventions, we conducted a needs assessment of all the PEM fellows. We administered a short questionnaire to assess their perceived barriers to achieving full scanning education (Table 1A). After the fellows identified the barriers at our institution, we categorized them into 6 broad categories on a fishbone diagram: knowledge, policy, environment, people, process, and accountability (Fig. 1). Then, we assembled a small group of fellows to discuss the cited barriers and for them to suggest potential interventions to address those barriers. The following key drivers were distilled from that discussion: confidence in skills, opportunities to scan, workload constraints, and clear expectations and metrics (Fig. 2).

**Table 1.** Assessments by PEM Fellows regarding Barriers to Using POCUS

A. Needs Assessment before Interventions What are the barriers to scanning educationally when working clinically? (select all that apply)	% of Fellows (N = 11 responses)
No interest—It’s not my thing.	9.1 (1)
Time it takes to do the scan (takes too long—negative impact on efficiency)	81.8 (9)
Haven’t figured out how to fit it into workflow.	72.7 (8)
Confidence in knowing how to use the machine to obtain and save images.	45.5 (5)
Don’t feel like there is value if the patient is getting a comprehensive radiology scan already.	27.3 (3)
B. Reassessment after interventions What are the barriers to scanning educationally when working clinically? (select all that apply)	% of Fellows (N = 14 responses)
No interest—It’s not my thing.	7.2 (1)
Time it takes to do the scan (Takes too long—negative impact on efficiency)	35.7 (5)
Volume of clinical tasks on shift—There is too much to do already.	100 (14)
Confidence in knowing how to use the machine to obtain and save images.	7.2 (1)
Confidence in interpreting images correctly.	42.9 (6)
Don’t know what indications to do educational scans for.	0 (0)
Don’t feel like there is value if the patient is getting a comprehensive radiology scan already.	21.4 (3)
Don’t know what the expectations are for POCUS (goal numbers, types of scans).	7.2 (1)
Haven’t figured out how to fit it into workflow.	35.7 (5)
Not enough POCUS-trained faculty to use POCUS effectively and safely (support).	42.9 (6)
POCUS is not a priority in our department.	14.2 (2)
C. Vote for the most effective interventions Which strategies have been effective in supporting educational scanning? (select all that apply)	% of Fellows (N = 14 responses)
Attending a scan session at least once a quarter (in quarters where you don’t have dedicated US time), so 3 additional times a year	100 (14)
Maintaining the scan counts dashboard	78.6 (11)
Going over scan numbers at COCs twice a year	21.4 (3)
Going over scan numbers in fellows’ meetings (occasionally)	14.3 (2)
Limiting the number of learners at the bedside (max. 3)	64.3 (9)
Attending Zoom QA to keep up with interpretation skills	21.4 (1)
Scanning with a co-fellow (whomever you’re working with clinically in the ED)	78.6 (11)
Trying to get at least 1 scan in when POCUS credentialed faculty are working clinically	50.0 (7)
Trying to scan on float/procedure shifts	92.9 (13)
Being oriented to POCUS expectations early in the first year	64.3 (9)

COC, clinical oversight committee; US, ultrasound.

**Fig. 3.**
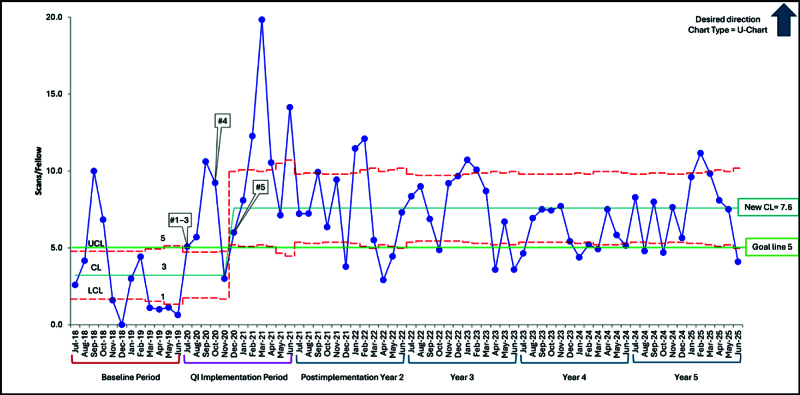
Total scans performed per fellow per month (U-chart of QI interventions). Initial baseline number of scans per fellow per month before QI intervention implementation was 3. Following QI project implementation, and after 8 successive points above the baseline centerline, a new centerline was identified at 7.6 scans per fellow per month in December 2020. The upper and lower control limits are shown in red dotted lines. The centerline (mean) is light blue. The goal line of 5 scans per fellow per month is indicated in green. The data labels (#1–5) reflect the interventions. CL, centerline; LCL, lower control limit; UCL, upper control limit.

In addition to the original ultrasound curriculum, the following inventions were implemented. The interventions are numbered to match the data labels on Figure 3 (overlapping interventions are grouped in time, and required interventions are marked with an * here):

- Orient first-year fellows regarding expectations (#1)*.
- Scanning during their “procedure” shift when they are present in the ED to look for scanning procedure opportunities while providing clinical care. The “procedure shifts” are 9-hour clinical shifts (#1).
- Seeking out their co-fellows or POCUS faculty when working clinically, especially those who may also be working in the ED at the same time to incorporate spontaneous scanning into workflow (#1).
- Reduce the number of learners at the bedside from 4 to 3 (#2)*.
- Create and maintain a scan count dashboard (#3)*.
- Attend a scan session at least once per academic quarter, supervised by POCUS fellowship–trained faculty during the entire 3 years of training (#4)*.
- Review scan numbers biannually at fellowship scholarly oversight committee meetings (#5)*.

**Fig. 4.**
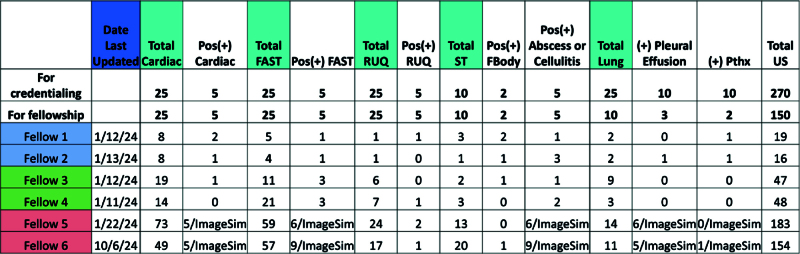
Condensed dashboard of PEM fellow scan counts (all fellows and applications not shown for brevity). Credentialing goals were based on ACEP guidelines in each application. Fbody, foreign body; Pos(+), positive (pathology seen on scans) or competency achieved in that module on ImageSim; Pthx, pneumothorax; RUQ, right upper quadrant; ST: soft tissue; US, ultrasound.

The POCUS director met with incoming first—year fellows in July 2020 to clarify expectations regarding 150 scans and to introduce a simple scan count monitoring dashboard. This dashboard was created on Google Excel (Mountain View, CA) listing each fellow’s initials in the rows and types of scans in each column. All the fellows are viewable on the same grid, so they can see how they are performing in relation to each other. The number of true positives is also recorded on the grid, as is competency achieved on ImageSim e-modules. The example shown is condensed and de-identified (Fig. 4). The dashboard count is verified by the POCUS director. The fellows were also required to present their scan numbers biannually at their Clinical Oversight Committee meetings. All scans that are saved in OsiriX and meet QA criteria are counted as a scan for the listed fellow. Multiple fellows can be credited with the scan if they are listed as being present for the scan.

## MEASURES

The primary outcome measure was the number of monthly scans performed by fellows in the ED. There was a month-to-month variation in the number of fellows in the ED because of their participation in clinical rotations outside the ED. Additionally, a fellow was not counted in the denominator if they were on maternity or paternity leave. Due to a variable number of fellows working clinically any given month, the number of scans each month is presented as an average. All fellows’ scans that met QA standards as verified by the POCUS director were counted (ie, both on supervised scan sessions and when working clinically). The secondary outcome measure was the total number of scans completed by third-year fellows by graduation. The baseline centerline was 3 scans per fellow per month, the equivalent of 90 scans per fellow during 3 years in the baseline period. The goal line was set at 5 scans per fellow per month, the equivalent of 150 scans per fellow during 3 years.

After implementation of the QI project and at the end of the study period, we sent a follow—up questionnaire to the fellows where we reassessed the barriers with more detailed questions than in the first needs assessment (Table 1B). In addition, the questionnaire asked the fellows, “Which strategies were most effective in practice to support educational scanning?” with the list of interventions described (Table 1C). We also queried any unintended consequences of the additional scanning requirement.

QI Macros (Excel Add-In, Denver, CO) was used for data analysis. QI methodology, including the Model for Improvement and statistical process control charts, was used to analyze the data and assess the impact of interventions. A U-chart is used to display the data due to scan count with a changing monthly denominator. We used standard statistical process control chart rules to determine centerline shifts (8 or more points at or above the centerline). The recommendations such as scanning on a procedure shift, scanning with a co-fellow, scanning with POCUS faculty when they are working clinically, and incorporating POCUS into workflow were difficult to pinpoint the exact time of execution on the U-chart, but were suggested at the beginning of the intervention period.

This initiative was reviewed by our institutional review board, and they determined that this initiative did not constitute human subjects research; as such, it was not under the oversight of the institutional review board. Squire 2.0 guidelines for QI reporting excellence were followed in this article.^18^

## RESULTS

### Needs Assessment before Interventions Implemented

As outlined in the fellow’s needs assessment, the top 3 barriers to achieving 150 scans per fellow during the 3-year fellowship included: (1) amount of time it takes to scan (81.8%); (2) inability to fit POCUS into workflow (72.7%); and (3) confidence in using the machine (45.5%) (Table 1A).

### Longitudinal Scanning

The U-chart displays the average number of scans done monthly per fellow (Fig. 3). The peaks represent most of the months when a second—year fellow was on their month-long ultrasound rotation, with a resultant larger volume of POCUS scans that month. There were 12 special cause variations within the 60 months. Two were in November and December due to the seasonal holidays in the United States, with limited scanning opportunities due to fewer clinical shifts and scan sessions, resulting in a lower number of scans. Additional special cause variations included time devoted to PEM faculty scanning for a different initiative (4 mo), time when POCUS faculty were away on holiday (2 mo), and 4 other months when fellows split their second—year rotation over 2 separate months or fewer fellows were on scan sessions.

**Fig. 5.**
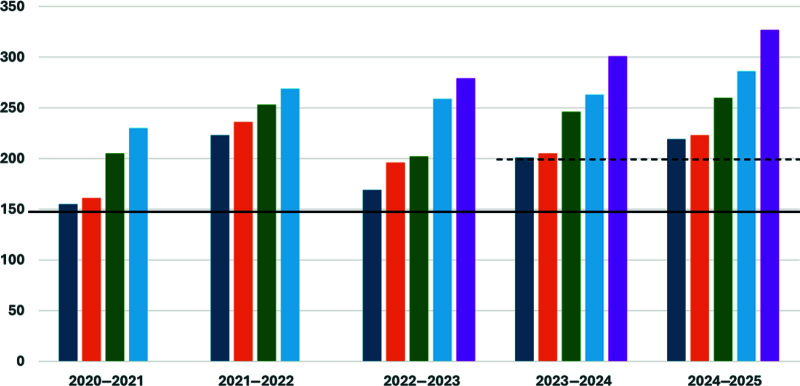
Total scans by graduating fellows of each academic year since 2020–2021 (study year). The solid black line represents the original target of 150 scans. For the graduation year 2023–2024 and onward, we increased the target to 200 scans (black dotted line).

The centerline was shifted in December 2020 to 7.6 scans per fellow per month, which forecasted 270 scans per fellow during 3 years. The impact has been sustained for 5 years since the initiation of the project. In the intervention year, all 4 of the graduating fellows met the threshold of 150 scans at graduation, which was the first time that was achieved since the start of the PEM POCUS program at our institution in 2012. Since then, all graduating fellows have met the threshold of 150 scans per fellow (range 201–327 scans) (Fig. 5).

When we reassessed the barriers that the fellows perceived at the end of the study, the number of tasks on shift was noted as the primary barrier by all the respondents (Table 1B). Regarding the most effective interventions, all 14 fellows who participated in the interventional changes to the curriculum cited the additional required longitudinal quarterly scanning as the most effective to support educational scanning. The next most impactful intervention, as described by the fellows, was trying to scan on procedure shifts (92.9%), scanning with co-fellows when working clinically (78.6%), as well as the creation of the scan count dashboard (78.6%) (Table 1C). None of the fellows reported unintended negative effects due to the additional requirement of a 3-hour scan session in an academic quarter. Additionally, the third-year fellows attained the numbers for credentialing per ACEP guidelines in additional ultrasound applications, such as Renal and Biliary, beyond the standard Soft Tissue, Bladder, FAST, and Cardiac applications.

## DISCUSSION

By instituting a new requirement of a supervised scan session every quarter for all fellows, creating a shared transparent scan count dashboard, and encouraging the fellows to scan together when working clinically and on procedural shifts, we surpassed our aim of increasing our fellows’ educational POCUS scanning. These interventions became hardwired into our system, such that the increase has been sustained for 5 years since the interventions were implemented. Notably, the results of our needs assessment varied from barriers cited in prior literature, emphasizing the importance of a systematic QI approach when planning interventions in one’s own institution. The graduating fellows attained the target of 150 scans per fellow despite variations in patient volumes during their fellowship and study period. Volumes significantly decreased in the first year of the COVID-19 pandemic and then were exceptionally high in 2021–2022 during respiratory surges. In addition, the number of POCUS fellowship–trained PEM faculty decreased during the study period: with 5 POCUS fellowship–trained PEM faculty in the baseline period, 4 in the study period, and 3 in the years following the study period. This lessened availability of scan sessions and highlighted the importance of increasing overall PEM faculty credentialing in POCUS to support trainee scanning. Despite these variables, our results surpassed the target we set for increased educational scanning by the fellows.

The success of the interventions to achieve more than 150 target scans is likely attributed to several key factors. First, the quarterly requirement of hands-on scanning provided the longitudinal reinforcement of both the mechanical and interpretative aspects of POCUS. On the procedure shifts, the fellows were not responsible for managing their own set of patients, and so they could scan when the clinical burden of tasks was decreased, directly addressing the most-cited barrier to educational scanning in the needs assessment. Scanning with a co-fellow addressed the lack of confidence identified as a barrier in scanning alone. The reassessment questionnaire showed strides in improved confidence in scanning, incorporating POCUS into the clinical workflow, and a decreased perception of time to do the scan as a barrier. Presumably, if one were more facile in using the machine and interpreting the images because of more frequent use, then the perception that POCUS takes “too long” is decreased. Additionally, peer benchmarking, as we did with the scan count dashboard, was an effective tool for improving performance outcomes among physicians.^19^

As described by the fellows, there were no unintended consequences of the interventions. The 3 hours to join 1 scan session a quarter came from their time when not doing other monthly rotation responsibilities. The fellows did not consider this new requirement as onerous, and it aligned with achieving their educational goals. The longitudinal opportunity for quarterly scanning was especially meaningful to senior graduating fellows who supplemented their scan numbers to meet ACEP credentialing criteria in additional POCUS domains. After the successful incorporation of our QI interventions, for the graduating class of 2024 onward, the number of required scans was raised to 200 (Fig. 5). For the next graduating class of 2026, the bar will be set at 250, as the fellows have demonstrated more than enough capacity to reach those parameters. There was no additional cost or time associated with the intervention as POCUS faculty continued their usual weekly scan sessions.

Future research should investigate whether these POCUS achievements during a PEM fellowship translate into practice as a PEM attending. Future investigations should work to identify effective strategies to facilitate continued scanning and maintenance of competency in POCUS following PEM fellowship graduation. This QI project highlighted the need to increase and maintain PEM faculty credentialing and expertise to support the PEM POCUS fellowship training. We are currently implementing a QI project to address that physician gap in our division.

## LIMITATIONS

The primary limitation is the generalizability of our work because of the strength of our POCUS program, including the number of PEM POCUS–trained faculty, availability of supervised scanning sessions, and well-developed QI processes. These resources may be difficult for other institutions to implement if there are constrained financial resources. Because POCUS training is a required element of every PEM fellowship program, programs with constrained resources or those that do not have a dedicated POCUS director can develop partnerships with Adult POCUS Programs to bridge the gap. Leveraging those liaisons can foster opportunities for continued scanning. Additionally, if there is limited time for POCUS faculty to offer continued supervised scanning, then fellows could be commissioned to scan together at least once a quarter, with senior fellows helping novice junior fellows. Another limitation of our work is that our image archiving database does not have an easy counting function, which introduces potential human error. The PEM POCUS faculty performing weekly QA could have missed noting a performed scan in the Exam Type section on OsiriX, and the system required the director to manually count the scans that met QA standards. However, that error would have resulted in an undercount.

## CONCLUSIONS

With our QI initiative, we achieved a 3-fold increase in PEM fellows’ POCUS scanning, with all graduating fellows surpassing the target of 150 scans, and most of them now, 5 years since the start of the study, reaching more than 250 scans. The most effective interventions included required quarterly supervised scanning, maintaining a visible and transparent scan count dashboard, and encouraging fellows to scan with each other when doing clinical work. These strategies can be incorporated by other PEM training programs, and it is hoped that they will be translated into practice by the next generation of PEM practitioners.

## ACKNOWLEDGMENTS

The authors thank Dr. Asha Payne for her feedback on the article.
